# A review of drowning prevention interventions in children and adolescents

**Published:** 2023-12

**Authors:** Zahra Asgari Tapeh, Azar Darvishpour

**Affiliations:** ^*a* Department of Nursing, Zeyinab (P.B.U.H) School of Nursing and Midwifery, Guilan University of Medical Sciences, Rasht, Iran. ^; ^*b* Social Determinants of Health (SDH) Research Center, Guilan University of Medical Sciences, Rasht, Iran. ^

## Abstract

**Background:**

Drowning is one of the leading causes of accidental death that has a significant impact on families, communities, and public health systems. Despite significant efforts made worldwide, the highest risk group for drowning is children aged 1-4 years and adolescents. This review aimed to identify risk factors and interventions to prevent drowning in children and adolescents.

**Methods:**

The present study is a systematic review. In order to collect the data, English articles available on Web of Science, Google Scholar, PubMed, and Scopus databases were reviewed from January 2000 to June 2023 with the keywords “Review”, “Drowning”, “Prevention”, “Children”, and “Adolescents”. Qualitative evaluation of the studies was done using evaluation tools recommended by the Joanna Briggs Institute. Data analysis was done by qualitative content analysis.

**Results:**

Among the 76 articles related to the subject of this research, some were excluded, and 29 articles were included in the final review. By analyzing the data, the risk factors of child drowning were in 5 main categories including “Unsafe environment” with 2 sub-categories, “Co-morbidities” with 2 sub-categories, “Demographic characteristics” with 3 sub-categories, “Substance Abuse” with 2 sub-categories, and “Environmental factors” with 2 sub-categories. Interventions to prevent drowning of children and adolescents were in 3 main categories including “Increasing knowledge and skills” with 2 sub-categories, “Restricting access to water” with 2 sub-categories, and “Securing the swimming pool and outdoor swimming environment” with 8 sub-categories.

**Conclusions:**

Drowning in children and adolescents is a multifactorial and challenging issue. Drowning prevention interventions identified in this study can be considered by officials and practitioners for policy-making and designing drowning prevention protocols for children and adolescents. Giving specific education to children, adolescents, parents, caregivers, and the public about risk factors and urgent interventions related to drowning, is the most important part of the multi-faceted approach to reducing fatal drownings.

**Keywords:**

Drowning, Prevention, Children, Adolescents, Review

